# I Choose Health. It is OncoLogical!—Informative and Educational Campaign Dedicated to the High School Students in Poland

**DOI:** 10.1007/s13187-023-02308-6

**Published:** 2023-05-12

**Authors:** Łukasz Moskal, Konrad Reszka, Krzysztof Szewczyk, Rafał Matkowski, Dawid Błaszczyk, Adam Maciejczyk

**Affiliations:** 1 Department of Oncology, Wroclaw Medical University, Wrocław, Poland; 2 Department of Surgical Oncology, Comprehensive Cancer Center, Wrocław, Poland; 3 Comprehensive Cancer Center, Wrocław, Poland; 4 Department of Radiation Oncology, Comprehensive Cancer Center, Wrocław, Poland

**Keywords:** OnkoLogika, High School Students, Cancer Awareness, Cancer Prevention, Educational Campaign

## Abstract

Cancer is the second most common cause of death in Poland and the number of new cases is expected to increase by 28% over the next 10 years. Despite modifications and expenditure growth in the Polish health care system, oncological treatment outcomes are lower comparing to the other European Union countries. Early preventative interventions are effective in reducing the total number of cancers and improving early detection. OnkoLogika is an educational campaign launched in 2016 by the Comprehensive Cancer Centre, aimed at improving cancer awareness. One hundred and twenty students from 25 high schools of the Lower Silesia region in Poland participated in the OnkoLogika program, which consisted of four-segment workshops containing pre-/post-tests, theoretical and practical parts within the project. The mean number of correct answers from the both tests improved after educational intervention (*p* < 0.001). Students’ knowledge increased, especially in relation to risk factors of breast cancer development (416.31% increase), HPV-related cancers (344.81% increase), risk factors and red flag signs of skin melanoma (120.31% and 99.05% increase respectively). Approx. 86% of participants were satisfied with the OnkoLogika with 14% of respondents being dissatisfied and 94% declared increased awareness about cancer prophylaxis. High schools students indicated insufficient time (250; 16.67%) and lack of details considering presented cancers (80; 5.33%) to be the major weaknesses of the program. Nevertheless, 94% of participants would recommend OnkoLogika to a friend. OnkoLogika promotes healthy lifestyle and helps acquire necessary knowledge about chosen cancers.

## Introduction

Cancer is the second most common cause of death in Poland with 185 thousand new cancer cases and 113 thousand deaths each year [1, 2]. Further, 43.1% of noncommunicable diseases premature deaths in 2016 were caused by malignancies [2]. It is estimated that in the next 10 years, the number of new cases will increase by 28% and cancer will become the leading cause of death in Poland [1].

Outcomes of oncological treatment in Poland are still unsatisfactory. EUROCARE-5 report showed that all cancers cumulative five-years survival rate for Poland is only 43% with the average for the EU27 at 54.6% [3]. This disparity may result from insufficient health care expenditure and restricted access to innovative cancer drugs, given the stringent criteria for placing innovative drugs on the reimbursement list, which significantly limits their availability in Poland [4].

The National Health Fund (NHF) spent USD 2.5 billion on cancer treatment in 2018, while only USD 42 thousand were spent on prophylaxis in 2015, which constitutes about 0.24% of NHF annual expenditure on health care services [5, 6]. In 2015, productivity loss due to cancer disease constituted 0.58% (USD 228.6 million) of Gross Domestic Product (GDP) in comparison to 0.59% (USD 238.8 million) in 2016 [7]. Moreover, the indirect costs of oncological diseases will increase to 1.30% of GDP in 2025 according to data presented by Polish Oncological Society [7].

Based on the data from the World Health Organisation (WHO), over 40% of all cancers can be prevented and many of them are curable if detected and treated early [8]. As reported by the Supreme Audit Office, only 21.3% of men and 33.6% of women participated in prevention programs in 2014, while in the years 2012–2015, expenditure on the national cancer control program decreased by 10% and were inappropriately allocated. [6]. As the costs of oncological treatment increase and the budget of the NHF is limited, the value of cancer education and prevention cannot be overstated.

It is considered that educational interventions dedicated to the high school students will improve cancer literacy and potentially increase effectiveness of cancer treatment in the future. Consequently, the students are targeted early because it is a perfect time frame for building up awareness about health behaviors [9, 10]. Since the risk of oncological diseases increases with age, development of health literacy among the young generation builds the basics for help-seeking behaviors later in life [11, 12].

However, there is limited evidence regarding the long-term effectiveness of oncological education among high school students [13].

While there are researches reporting poor knowledge of polish high school students about cancer risk factors, there are some educational programs intended to fulfill this gap—mainly based on community activity of medical students and dedicated to a limited number of students [14].

Therefore, despite various modifications to high schools curricula in recent years, the cancer prevention literacy level among polish students is still unsatisfactory [14]. Further, the lack of regular physical activity in schools hampers efforts of medical professionals and significantly affects development of chronic diseases according to the US Centers for Diseases Control and Prevention (CDC) [15].

In 2016 the Wrocław Comprehensive Cancer Centre with the financial support from Lower Silesian Self-Government created an informative and educational campaign for the population of Lower Silesia, including a curriculum for high school students. The program titled OnkoLogika has following objectives: (a) to educate Lower Silesians about chosen cancers, (b) broaden the cancer awareness of Lower Silesians, (c) increase the number of preventive medical examinations, (d) develop pro-preventive attitude. Additionally, the project was aimed to dispel the myths about cancer and raise awareness that cancers detected early can be treated more effectively.

To achieve our goals, we used a mix of different methods and instruments. Each of program activities was connected with the media briefing and expert meetings. Involvement of mass media in promotion of healthy life-style is beneficial and can reduce the number of obstructive modifications in health-related behaviors across society [16]. Moreover, impact of NGOs on health advocacy and formation of adequate health policies, both in urban and rural areas is visible and positive [17, 18]. Thus, conferences were divided into two parts: 1) the local media briefing—introduction to the campaign, 2) expert conference with discussion between health care professionals and members of the local community. Further, to spread the knowledge about oncological diseases and methods of prevention across the region, not only regional media, but also the organizations fighting against cancer and promoting pro-preventive attitudes were engaged.

In order to increase effectiveness and produce long-lasting effect of educational interventions, involvement of local communities is crucial and affects various determinants of health [19]. Thanks to coverages prepared by journalists participating in the workshops, the local community could additionally benefit from the curriculum and oncological knowledge we presented. What is more, the leaflets and posters regarding aims of the OnkoLogika were prepared and distributed during press conferences and promotional action titled “day of health” to reach out to members of the community that could not participate in the events.

Our actions were not limited to in-person activities. Social media is extremely popular, especially among the younger generation and plays an important role in data dissemination [20]. Consequently, to improve the reach of OnkoLogika campaign, the website and Facebook page of the project were created, as a source of knowledge regarding the campaign and cancer prevention. Short, interactive webinars and expert movies were made by medical experts from Comprehensive Cancer Centre to further disseminate information. Moreover, in order to raise oncological awareness in the communities we could not visit in person, educational materials were sent to more than 100 high schools across the Lower Silesia, and from the beginning of the project 12 billboards promoting healthy lifestyle and screening test were placed in the cities of the region.

The educational campaign was conducted in-person during the 2017–2019 school years. Since 2019, amid the COVID-19 pandemic most of the campaign activities were held online. This manuscript addresses the results of this project and discusses the usefulness and effectiveness of the OnkoLogika curriculum for education of the young generation.

## Materials and Methods

### Study Design and Materials

All high schools of Lower-Silesia were invited to participate in our study. The schools were chosen on first-come-first-service basis. Finally, twenty-five high schools participated in OnkoLogika. All students from chosen schools were asked to take part in our program. Participation was voluntary; however, the written informed consent of parents/guardians was required for minors.

Approximately 120 students of each school, aged 16–19 years took part in workshops with a total number of 3.000 participants. The curriculum was based on workshops conducted by trained psychologists and oncologists, dedicated to the subject of the most common, preventable or easily treatable when detected early cancers: breast, lung, cervical cancer and skin melanoma.

Each session consisted of 4 parts: a) pre-test, b) theoretical part, c) practical part and d) post-test (Table 1). 1,500 participants returned correctly fulfilled surveys, which constituted the final study group (rr = 50.00%).

**Table 1:** Sample workshop description

1. Pre-test (5 min.) – diagnoses level of students’ knowledge in four types of cancer (breast, cervix, lung and skin melanoma)	2. Theoretical part (25 min.) – interactive lectures with the most important topics, including: specific types of cancer, risk factors, alarming symptoms, self-examination, importance of prevention, possible treatment, impact of healthy lifestyle
3. Practical part (90 min.) – group exercises titled:	
- “I know HIM inside out” – four students’ groups are preparing a poster about chosen cancer (breast, cervix, skin, lung) with scrapbooking method	
- “To euchre the cancer” – based on the pictionary method, four students’ groups are guessing slogans being simultaneously risk factors for cancer development	
- “I check the ground” – the students learn about self-examination on anatomical models	
4. Post-test (15 min.) – determines the level of students’ knowledge in four types of cancer (breast, cervix, lung and skin melanoma) after our intervention	

According to polish law, the IRB approval was not required for our study. Collected data was stored and analyzed by the Wrocław Comprehensive Cancer Centre according to the European Parliament and Council General Data Protection Regulation and institutional privacy policy [21, 22].

### Variables

Pre- and post-workshop surveys consisted of 9 multiple-choice questions, including the most common cancers among men and women, typical age range, risk factors for cancer development, alarming symptoms of cancer and knowledge about chosen types of cancer (breast, lung, cervical cancer and skin melanoma). Moreover, participants were asked to evaluate workshops and presenters according to a four-level Likert scale (“very satisfied”, “satisfied”, “unsatisfied”, “very unsatisfied”). At the end of the survey, participants were asked to indicate strengths and weaknesses of the project in the open-ended questions. Surveys did not include inquiries considering socio-demographic data of participants. Due to COVID-19 pandemic restrictions, it was not possible to collect surveys after 6-months, as it was planned initially.

### Statistical Analysis

In the statistical analysis, the dependent t-test was used to assess the relationship between the studied means. Statistical analysis was performed using Statistica v.12.5 (StatSoft).

## Results

Statistical analysis was conducted on a sample size of 1,500 correctly completed questionnaires. The number of correct answers for each of 9 questions from pre- and post-test was assessed (Table 2), with mean scores of 709 (47.3%) and 1275 (85%) respectively. The mean number of correct answers significantly improved after educational intervention (*p* < 0.001, Table 3).

**Table 2:** Evaluation of students’ knowledge on the group of 1,500 respondents

No	Text of the question	Number of correct answers (%)		Knowledge increase (%)
		Pre-test	Post-test	
1	What are the most common types of cancer in Poland?	1356 (90.4%)	1422 (94.8%)	4.87
2	What are the most common types of cancer among the men in Poland?	1158 (77.2%)	1389 (92.6%)	19.95
3	What is typical age range for breast cancer?	257 (17.13%)	1327 (88.47%)	416.34
4	Is birth control associated with higher risk of breast cancer?	547 (36.47%)	1402 (93.47%)	156.31
5	By how many percent physical activity reduces risk of breast cancer?	497 (33.13%)	1115 (74.33%)	124.35
6	What are the risk factors for lung cancer?	1256 (83.73%)	1451 (96.73%)	15.53
7	What are alarming symptoms of melanoma?	524 (34.93%)	1043 (69.53%)	99.05
8	What type of cancer can be triggered by HPV?	270 (18%)	1201 (80.07%)	344.81
9	What are risk factors for melanoma?	512 (34.13%)	1128 (75.2%)	120.31

**Table 3:** Evaluation of workshops

Number of correct answers	n	Mean	SD	t-cal	df	*p*
Before intervention	9	708,5556	427,2974	-4,86,297	8	0,000,626
After intervention	9	1275,333	154,4838			

While more than 90% (1,356 individuals) of students were able to indicate the most common malignancies in Poland and approximately 84% (1,256 individuals) knew the risk factors related to the development of lung cancer, only one-third of respondents correctly indicated predisposing factors for breast cancer (547; 36.5%) and skin melanoma (512; 34.1%), as well as its red flag symptoms (524; 34.9%). Only 18% (270 individuals) of students correctly indicated cancers related to HPV-infection.

Post-test knowledge increases were observed for risk factors connected with breast cancer development (547; 36.47% versus 1,402; 93.47%), HPV-related cancers (270; 18% versus 1,201; 80.07%), risk factors and red flag signs of skin melanoma (512; 34.13% versus 1,128; 75.2% and 524; 34.93% versus 1,043; 69.53% respectively), when compared to pre-test scores.

Upon completion of the project, most (1,290 individuals; 86%) participants were satisfied with the OnkoLogika curriculum (915; 61% “very satisfied”, 375; 25% “satisfied”), while only 14% (210 individuals) of respondents were dissatisfied (195; 13% “unsatisfied”, 15; 1% “very unsatisfied”). Ninety-seven percent of participants (1,455 individuals) indicated that information presented during the project was comprehensible, and 96% (1,440 individuals) rated the quality of the workshops highly. Ninety-five percent of participants stated that OnkoLogika broadened their oncological knowledge, 97% (1,455 individuals) considered the knowledge and skill acquired during the workshops to be useful in the future and 94% (1,410 individuals) declared increased awareness in cancer prevention. Finally, 94% (1,410 individuals) of participants indicated that they would recommend OnkoLogika to a friend.

The greatest strengths of the project in the opinion of respondents were group work (327; 21.8%), posters presentation (280; 18.67%), interactive lectures (230; 15.33%), workshops topics (153; 10.2%), methods of knowledge sharing (150; 10%) and commitment of project’s members (72; 4.8%). On the other hand, the biggest weaknesses were insufficient time (250; 16.67%) and lack of details considering presented cancers (80; 5.33%).

## Discussion

OnkoLogika is a campaign based on two pillars—promotion and education with a wide range of educational interventions dedicated to the community of Lower Silesia. Nevertheless, the curriculum dedicated to high school students constitutes the core activity of the program.

While the long-term effect of educational interventions is uncertain, there is a plethora of evidence that they increase cancer literacy in a short-term follow-up [13]. In the analysis of 12 educational programs prepared by Khadija Al-Hosni et al. the duration of follow-up for these interventions was limited to the same academic year. Thus, long-lasting effects of these interventions cannot be concluded [13].

Nevertheless, there are analyses reporting development in cancer literacy knowledge levels as a result of educational interventions. [13]. Recent study revealed a significant increase in cancer literacy levels immediately after the short educational intervention based on a 30- to 45-min PowerPoint presentation, dedicated to Kentucky high school students aged 15–18 [23]. Unfortunately, there are many limitations of this study, mainly due to its cross-sectional character [23].

A 2018 study of Iranian students' knowledge about skin cancer risk factors and preventive behaviors used educational intervention based on the PRECEDE model, which is a systematic process for planning, presenting, and evaluating health promotion programs for defined populations [24]. While the strong point of this analysis is presence of the control group, it should be noticed that gender was restricted and carried out only on male students. Four months after the intervention, the experimental group showed a significant increase in the knowledge, attitude, self-efficacy, enabling factors, social support, and skin cancer preventive behaviors compared to the control group [24]. All mentioned studies are in line with our paper.

Despite the fact that some cancers are preventable, modifications of lifestyle and pro-preventive attitude are frequently started too late [25]. Further, most of the funding and research available focuses on treatment rather than prevention [25]. Therefore, it is important to raise awareness and encourage all inhabitants of Lower Silesia to pay attention to the necessity of carrying out preventive medical examinations. With that necessary knowledge, cancer can be detected early and treated effectively. By targeting high school students, habits and beliefs can be changed early established.

The program catered to different learning styles, with group work, lectures and written material all being rated as valuable modes of content delivery. E.g. different practical activities, like “I know HIM inside out” or “I check the ground”, develop students’ horizons in terms of available screening tests and self-examination.

This study shows that whilst general knowledge about the most common cancers in Poland is acceptable, knowledge about risk factors of oncogenesis as well as red flags signs and symptoms is unsatisfactory. Consequently, this part of education was highlighted in OnkoLogika curriculum.

Further work is already planned to provide workshops for teachers to nurture the positive effects of the project through the provision of knowledge and resources and facilitating workshops themselves. In the next step, the long-term follow-up survey will be implemented in order to provide evidence about the long-term retention of the information in the participants.

### Limitations

Despite its comprehensive, innovative character, our study has several limitations. First of all, there was no control group, thus it is not possible to assess the putative impact of other educational campaigns that might have occurred in the same timeframe.

Secondly, due to lack of follow-up, the impact of our one-time training cannot be evaluated in a long-term perspective. Consequently, the long-term retention of the information in the participants remains uncertain. Moreover, the increase of high school students’ knowledge about chosen cancer may not be connected with the increased number of early detected oncological diseases. As the time is needed for students to reach an adequate age to enter screening programs, the real effectiveness and impact of our study can be observed in the following decades.

Another serious limitation is the lack of participants' characteristics. Pre- and post-test did not include questions considering socio-demographic data. As all the high school students were invited to take part in educational program and participation was voluntary, the study group is not representative.

Finally, this exploratory study should be interpreted cautiously and in context with its limitations. As a cross-sectional study of a convenience sample, the results may not be generalizable.

## Conclusion

Health literacy can be developed among the adolescents through educational interventions. OnkoLogika may help students acquire necessary knowledge about chosen cancers necessary to make health-oriented decisions in the future.

The project promotes a healthy lifestyle and can aid health-seeking behaviors. Our program met the expectations of the young generation. We suggest that high schools’ curricula incorporate evidence-based cancer education modules like OnkoLogika.

## Data Availability

The data reported in this manuscript have not been previously published and/or were not collected as part of a larger data collection (at one or more points in time). Findings from the data collection have not been reported in separate manuscripts. All data and materials support published claims and comply with field standards.

## References

[1] Poland. Burden of Cancer (2020) Cancer Country Profile 2020. WHO Europe. Available at: https://cdn.who.int/media/docs/default-source/country-profiles/cancer/pol-2020.pdf?sfvrsn=884da736_2&download=true. Accessed 28.03.2021

[2] Polish Society of Oncology et al (2017) Cancer Control Strategy for Poland 2015–2024. Available at: http://www.walkazrakiem.pl/sites/default/files/library/files/polish_cancer_control_strategy_0.pdf. Accessed 28.03.2021

[3] European Cancer Registry Based Study on Survival and Care of Cancer Patients (2008) Eurocare 5 Survival Analysis 2000 - 2007. Available at: https://w3.iss.it/site/EU5Results/forms/SA0007.aspx. Accessed 28.03.2021

[4] EY Poland (2015) Access to innovative cancer drugs in Poland in comparison with selected European Union countries and Switzerland. Report ordered by Young People Oncology Foundation Alivia and prepared by EY Poland. Available at: https://www.alivia.org.pl/raport2015/05_05_2015_Raport_wersja_EN.pdf. Accessed 28.03.2021

[5] Narodowy Fundusz Zdrowia (National Health Fund) (2020) Wydatki na onkologię w latach 2013–2018. Available at: https://www.nfz.gov.pl/download/gfx/nfz/pl/defaultaktualnosci/370/7590/1/wydatki_onkologia.pdf. Accessed 11.04.2021

[6] Najwyższa Izba Kontroli (Supreme Audit Office) (2016) Profilaktyka Zdrowotna w Systemie Ochrony Zdrowia, Informacja o wynikach kontroli. KZD.430.007.2016. Nr ewid. 211/2016/P/16/054/KZD. Available at: https://www.nik.gov.pl/plik/id,13788,vp,16224.pdf. Accessed 11.04.2021

[7] Holecki Tomasz, Węgrzyn M, Frączkiewicz-Wronka A, Sobczyk K (2020). Oncological Diseases and Social Costs Considerations on Undertaken Health Policy Interventions. Int J Environ Res Public Health.

[8] World Health Organization (2008) Cancer Control: Knowledge Into Action: WHO Guide for Effective Programmes. Geneva. Available: https://apps.who.int/iris/bitstream/handle/10665/43467/9241546999_eng.pdf;jsessionid=D585C2168F5BCE17D41CB08634F299DE?sequence=1. Accessed 11.04.2021

[9] Fondjo LA, Owusu-Afriyie O, Sakyi SA, Wiafe AA, Amankwaa B, Acheampong E, Ephraim RKD, Owiredu WKB (2018) Comparative assessment of knowledge, attitudes, and practice of breast self-examination among female secondary and tertiary school students in Ghana. Int J Breast Cancer 7502047–7502010. 10.1155/2018/7502047 10.1155/2018/7502047 PMC6091363 30151285

[10] Barros Ana, Moreira L, Santos H, Ribeiro N, Carvalho L, Santos-Silva F (2014). “Cancer: educate to prevent”: high-school teachers, the new promoters of cancer prevention education campaigns. PLoS One.

[11] Kyle RG, Nicoll A, Forbat L, Hubbard G (2013). Adolescents’ awareness of cancer risk factors and associations with health related behaviours. Health Educ Res.

[12] Hubbard G, Stoddart I, Forbat L, Neal RD, O’Carroll RE, Haw S, Rauchhaus P, Kyle RG (2016). School-based brief psychoeducational intervention to raise adolescent cancer awareness and address barriers to medical help-seeking about cancer: a cluster randomised controlled trial. Psychooncology.

[13] Al-Hosni K, Chan MF, Al-Azri M (2021). The Effectiveness of Interventional Cancer Education Programs for School Students Aged 8–19 Years: a Systematic Review. J Cancer Educ.

[14] Rucinska M, Sroda R, Wilk O, Saied A, Miloszewski J, Sugajska A, Osowiecka K (2021). Polish High School Students' Knowledge about Cancer. Int J Environ Res Public Health.

[15] Booth Frank W, Roberts CK, Laye MJ (2012). Lack of exercise is a major cause of chronic diseases. Compr Physiol.

[16] Wakefield MA (2010). Use of mass media campaigns to change health behaviour. Lancet.

[17] Piotrowicz M, Cianciara D (2013). The role of non-governmental organizations in the social and the health system. Przegl Epidemiol.

[18] Gellert GA (1996). Non-governmental organizations in international health: past successes, future challenges. Int J Health Plan Manag.

[19] Haldane V, Chuah FLH, Srivastava A (2019). Community participation in health services development, implementation, and evaluation: A systematic review of empowerment, health, community, and process outcomes. PLoS One.

[20] Stellefson M, Paige SR, Chaney BH, Chaney JD (2020). Evolving Role of Social Media in Health Promotion: Updated Responsibilities for Health Education Specialists. Int J Environ Res Public Health.

[21] European Union Parliament and of the Council (2016) Regulation (EU) 2016/679 of the European Parliament and of the Council of 27 April 2016 on the protection of natural persons with regard to the processing of personal data and on the free movement of such data, and repealing Directive 95/46/EC (General Data Protection Regulation). Available at: https://eur-lex.europa.eu/eli/reg/2016/679/oj. Accessed 29.08.2022

[22] Wrocław Comprehensive Cancer Centre Privacy Policy. Available at: http://bip.dco.com.pl/obowiazek-informacyjny-oraz-obowiazek-prawa-pacjenta-do-kontroli-swoich-danych-i-uzyskania-informacji-o-zasadach-przetwarzania-jego-danych-osobowych/. Accessed 29.08.2022

[23] Hudson L, Samons KM, Dicken HE (2021). A Brief Educational Intervention Enhances Basic Cancer Literacy Among Kentucky Middle and High School Students. J Cancer Educ.

[24] Jeihooni AK, Moradi M (2019). The Effect of Educational Intervention Based on PRECEDE Model on Promoting Skin Cancer Preventive Behaviors in High School Students. J Cancer Educ.

[25] Colditz GA, Wolin KY, Gehlert S (2012). Applying what we know to accelerate cancer prevention. Sci Transl Med.

